# Changing geographic ranges of ticks and tick-borne pathogens: drivers, mechanisms and consequences for pathogen diversity

**DOI:** 10.3389/fcimb.2013.00046

**Published:** 2013-08-29

**Authors:** Nick H. Ogden, Samir Mechai, Gabriele Margos

**Affiliations:** ^1^Zoonoses Division, Centre for Food-borne, Environmental and Zoonotic Infectious Diseases, Public Health Agency of CanadaSaint-Hyacinthe, QC, Canada; ^2^Faculté de Médecine Vétérinaire, Departmeté de Médecine Vétérinaire, Université de MontréalSaint-Hyacinthe, QC, Canada; ^3^Department of Veterinary Sciences, Institute for Infectious Diseases and Zoonoses, Ludwig-Maximilians-University Munich, and German National Reference Centre for Borrelia, Bavarian Health and Food Safety AuthorityMunich, Germany

**Keywords:** range change, invasion, Ixodes, Lyme, genetic diversity

## Abstract

The geographic ranges of ticks and tick-borne pathogens are changing due to global and local environmental (including climatic) changes. In this review we explore current knowledge of the drivers for changes in the ranges of ticks and tick-borne pathogen species and strains via effects on their basic reproduction number (*R*_0_), and the mechanisms of dispersal that allow ticks and tick-borne pathogens to invade suitable environments. Using the expanding geographic distribution of the vectors and agent of Lyme disease as an example we then investigate what could be expected of the diversity of tick-borne pathogens during the process of range expansion, and compare this with what is currently being observed. Lastly we explore how historic population and range expansions and contractions could be reflected in the phylogeography of ticks and tick-borne pathogens seen in recent years, and conclude that combined study of currently changing tick and tick-borne pathogen ranges and diversity, with phylogeographic analysis, may help us better predict future patterns of invasion and diversity.

## Introduction

Change in geographic range is one process whereby infectious diseases emerge or re-emerge (Kilpatrick and Randolph, 2012). Many ticks and tick-borne diseases are of significance for human or animal health, so professionals in these fields have a keen interest in being able to identify, understand, and predict changes in their geographic ranges. Ticks and tick-borne pathogens that are increasing their geographic ranges are effectively invasive species and the processes of invasion, consequences for their genetic diversity, and their potential impact on the natural ecosystems that they invade remain mostly unstudied. Examples of ticks and tick-borne pathogens that have changed their range in recent decades include northward expansion of *Ixodes scapularis* and *I. ricinus* and the pathogens they transmit (Lindgren et al., 2000; Ogden et al., 2009; Léger et al., 2013; Medlock et al., 2013), as well as *Rhipicephalus* and *Amblyomma* spp. ticks (reviewed in Léger et al., 2013). Here we review the processes involved in range changes of ticks and tick-borne pathogens, and their consequences for genetic diversity of ticks and tick-borne pathogens, which in turn can have importance for understanding the ecology, pathogenicity and diagnostic detectability of tick-borne pathogens (e.g., Ogden et al., 2011). We investigate two epidemiological/ecological processes involved in range change: (i) the factors involved in the maintenance of tick-borne pathogen transmission cycles that may or may not permit expansions of ticks and tick-borne pathogen populations, and may or may not permit them to become endemic once they arrive in new locations; and (ii) the mechanisms whereby ticks and tick-borne pathogens are dispersed from locations where they are endemic allowing invasion of new, ecologically-suitable locations. Then we review possible expected consequences of these processes for the diversity of tick-borne pathogens (using the Lyme borreliosis group of spirochaetes as an example), and how we would expect them to be reflected in the phylogeography of ticks and *Borrelia burgdorferi* sensu lato (s.l.: the agent of Lyme disease, also called Lyme borreliosis). We show that many current hypotheses are supported by observations, which gives confidence that in the future we may, by combined study of the ecology of tick and tick-borne pathogen invasion, their genetic diversity and their phylogeography, be better able to predict future patterns of invasion and diversity.

## Processes involved in tick-borne pathogen maintenance

The basic reproduction number (*R*_0_) is an index of the rate of reproduction of a species or strain under the conditions occurring at a particular point in space and time. Therefore, *R*_0_ provides an index of the fitness of species and strains at locations where they occur, or in new locations into which ticks and tick-borne pathogens are invading. In this section we review the key variables affecting the basic reproduction number (*R*_0_) of tick-borne pathogens using the simplified equation of Randolph (1998):

$$R_{0}=\frac{Nf\beta_{V-T}\beta_{T-T}\beta_{T-V}p^{n}F}{H(r+h)}$$

Where *N* = number of tick vectors, *H* = number of susceptible reservoir hosts, β_*t*−*v*_, β_*t*−*t*_, β_*t*−*v*_ = pathogen transmission coefficients [respectively tick-to-vertebrate host, tick-to-tick (transtadially or transovarially) and vertebrate host-to-tick], *p* = vector daily survival probability, *n* = development duration for ticks (and *p*^*n*^ = interstadial survival rate), *r* = daily rate of loss of infectivity of host, *h* = host daily mortality rate, *f* = probability tick feeds on an individual of a host species, *F* = tick birth rate.

### *N*, the density of ticks

Tick density must be above a threshold to support tick-borne pathogen transmission cycles, and the higher the density of ticks, the more efficient transmission cycles are likely to be (Norman et al., 1999; Ogden et al., 2007). Tick density has two principal determinants: density of hosts (be they reservoir competent hosts or not) and tick mortality rates. In any one location, host density is in turn determined by the biotic and abiotic features of the community at that location (Begon et al., 2005). These features will also partly determine the range of host species, which may have consequences for pathogen transmission (see subsequent sections) and for rates of on-host tick mortality. While many tick species of importance for human and animal health are exophilic host generalists to a greater or lesser degree (e.g., *I. ricinus*, *I. persulcatus* and *I. scapularis*), some ticks are host specialists that are often nest-living (nidicolous, e.g., *I. trianguliceps, I. muris*). The density of hosts for the latter, the specialists, will in most cases be lower than for the former in most locations. Both feeding ticks and free-living ticks (i.e., those undergoing development from one instar to the next, those in diapause or those questing for a host) suffer mortality. Apart from host densities, rates of mortality of ticks that are not feeding on hosts are likely the main determinant as to whether a particular habitat is suitable for tick population invasion, establishment and maintenance. The capacity of a habitat to provide non-feeding ticks with refugia from extreme temperatures, desiccation, drowning or perhaps predation, within the limits provided by climate, will determine the mortality rates of ticks in that habitat (Lindsay et al., 1995, 1998; Ogden et al., 2006a). Ambient temperature also acts as a determinant of mortality rates of ticks that interplays with habitat qualities. The colder the climate the longer is the tick's lifecycle and, given constant daily, per-capita mortality rates, the less likely a larval tick is to survive to be a mated adult (Ogden et al., 2005). Below a threshold of temperature conditions tick populations will die out, or fail to invade. However, what that threshold is precisely, for a particular habitat, will depend on the mortality rates of the ticks in that habitat (Ogden et al., 2005, 2006a). It should be noted that temperature-independent (frequently daylength-dependent) behavioral or developmental diapause (Gray, 1991) occurs in the lifecycle of some tick species, in which case the duration of the lifecycle may be less influenced by temperature. In habitats that support off-host survival of ticks, host grooming (Levin and Fish, 1998) and effects of the innate and acquired immune responses of the host (which may of course be linked) are important causes of tick mortality (Randolph, 1979; Wikel, 1999; Ogden et al., 2002a,b). Mortality due to host grooming and acquired resistance are density-dependent in many species (Randolph, 1979; Levin and Fish, 1998; Goodwin et al., 2001; Shaw et al., 2003), the latter due to density-dependent activation of cell-mediated acquired resistance (Dizij and Kurtenbach, 1995; Ogden et al., 2002b), and may be a key mechanism for density-dependent regulation of tick populations (Randolph, 1998). Different host species may have a greater or lesser capacity to groom or express innate or acquired resistance so the host species range and relative abundance will determine the overall proportion of ticks that survive feeding (Dizij and Kurtenbach, 1995; Keesing et al., 2009). Thus, climatic conditions and habitat (acting together on tick mortality) as well as host range and abundance are all key determinants of range spread for ticks and tick borne diseases by acting on tick density.

### *f*, the probability that a tick feeds on an individual of a reservoir competent host species

The probability that a tick finds an individual host of a species that is a competent reservoir depends on the range and relative densities of these two types of host species (Tsao, 2009). This is effectively the theory behind the “dilution effect” of biodiversity (Ostfeld and Keesing, 2000; Dobson et al., 2006; Keesing et al., 2006), and although the effect of biodiversity *per se* as an inhibitor of tick-borne disease transmission in all circumstances is under question (Wood and Lafferty, 2013), high abundance of reservoir incompetent hosts in some circumstances likely inhibit transmission cycles (Ogden and Tsao, 2009; Tsao, 2009), for example when pathogens display host associations as seen for *Borrelia* species in Europe (Kurtenbach et al., 2002, 2006). Thus, rates at which ticks find a reservoir-competent host are largely determined by the composition of the host community, and the relative densities of reservoir host species.

### Transmission coefficients β_*T*−*V*_, β_*T*−*T*_, β_*V*−*T*_

Transmission coefficients (i.e., the efficiency) of transstadial transmission (β_*t*−*v*_ and then β_*v*−*t*_) and (if it occurs) transovarial transmission (β_*t*−*v*_ and then β_*t*−*t*_) are functions of the pathogen-tick interaction and key determinants of the basic capacity of the tick-borne pathogen to invade: if a tick-borne pathogen is poorly adapted to ticks in regions in which it is invading, these transmission coefficients will be lower, reaching zero if the vector is completely incompetent. Therefore, the geographic occurrence of competent tick vector species absolutely impacts the geographic range of tick-transmitted pathogens, although genetic change by random mutations can mean that tick-borne pathogens become adapted to transmission by new tick vector species (Margos et al., 2013). This is, of course, not a consideration where ticks and the pathogens they transmit are invading together or sequentially.

Whether or not tick-to-host transmission occurs (i.e., whether or not a host can acquire infection), depends on the innate susceptibility of individuals of the host community to infection, i.e., the capacity of the innate immune response of the host to kill the pathogen at the point of infection (Kurtenbach et al., 2006). While tick saliva contains many components that may inhibit the innate immune response, some functions are not affected and different host species may be differentially susceptible to different tick-borne pathogen species or strains (Kurtenbach et al., 2002). Host-to-tick transmission coefficients depend on the capacity of the pathogen to multiply in the host and disperse widely within the skin or blood of the host from which they can be transmitted to uninfected feeding ticks. This is a function of the capacity of the pathogen to evade the host innate and acquired immune responses. In general greater multiplication corresponds with greater transmission coefficients, but higher rates of pathogen multiplication in the host may result in a “cost” in terms of host mortality rates (Ogden et al., 2007 and see below).

Host-to-tick transmission coefficients are usually highest in early acute stages of infection, and then decline to low levels (assuming the pathogen is capable of more long-term immune evasion and persistence in the host) or to zero if host immunity is complete (Ogden et al., 2007). Some strains of *B. burgdorferi* s.s. are, however, capable of causing infections of wild rodents that result in persistently high host-to-tick transmission coefficients (Hanincová et al., 2008). The degree to which infecting and infection-acquiring instars (usually nymphs and larvae respectively) feed sequentially on hosts within a short enough time period determines whether infection-acquiring instars feed on hosts during the acute rather than the post-acute/chronic phases of infection, or on hosts that have recovered from infection (Ogden et al., 2007). Thus, the degree to which these tick instars are seasonally active at the same time (Randolph et al., 1999; Gatewood et al., 2009) is a determinant of the “average” host-to-tick transmission coefficient from the reservoir host population. The more persistent acute infections are though (i.e., the lower is *r*), the less impact tick seasonality will have.

Co-feeding transmission, where it occurs, may augment coefficients of host-to-tick transmission associated with systemic host infections (Randolph et al., 1996). The occurrence of co-feeding transmission depends on the capacity of pathogens to be transmissible by this route, and for individual host species to support co-feeding transmission. It also depends on patterns of coincident feeding of infecting instars and infection-acquiring instars on the same host at the same time, and therefore on seasonal activity patterns of the ticks (Randolph et al., 1999; Gatewood et al., 2009). Thus, overall the potential transmission coefficients for a particular pathogen at a particular location will depend on (i) the degree of competence of the vectors present, (ii) the composition of the host community and the relative densities of different reservoir host species, as well as (iii) the effects of seasonal tick activity on β_*v*−*t*_ and co-feeding transmission. Genetic change of the tick-borne pathogens can result in changes to vector and host competence, as well as the dynamics of host infections that determine β_*v*−*t*_.

### *P*^*N*^ the interstadial tick survival rate

The main determinants of interstadial survival are the daily per-capita mortality rates of engorged ticks in the environment, the duration of the development period, and the time it takes for a moulted tick to find a host. Daily per-capita mortality rates are determined by the qualities of habitat that determine mortality rates of ticks when off-host as discussed above. Development duration is determined largely by ambient temperature; the warmer the temperature, the faster is the development and the lower is the total proportion of ticks that will die (reviewed in Ogden et al., 2005). Microclimate (i.e., habitat-modified climate) affects tick activity which, combined with host densities, affects the rate at which ticks find a host (Ogden et al., 2005). In addition, interstadial survival rate will depend on the quality and quantity of the tick's last meal, which determines the fat reserves on which the moulted tick relies for host seeking (Randolph et al., 2002). In turn this depends on the host innate and acquired resistance to ticks: ticks that feed on hosts expressing resistance may not die but feed less successfully (Dizij and Kurtenbach, 1995; Ogden et al., 2002a). There is a roughly quadratic relationship between temperature and host seeking activity with activity inhibited at low and high temperatures (Vail and Smith, 1998, 2002). Low relative humidity at one extreme, and intense rainfall at the other are inhibitory of host-seeking activity (Randolph, 1997; Vail and Smith, 1998, 2002) so rainfall frequency and intensity can also have a quadratic relationship with host-seeking activity. Interstadial tick survival rate therefore depends on a combination of climate, habitat and host density.

### *F*, the tick birth rate

Tick birth rate depends on the proportions of adult female ticks that successfully mate, which is determined by the densities of suitable hosts (as female ticks must feed to reproduce), and the fecundity of individual female ticks, which depends on the quality and quantity of the female tick's meal. The latter will be determined by the community of adult tick hosts and their innate and acquired immune responses to the ticks (reviewed in Ogden et al., 2005). Thus, the tick birth rate will depend on the densities and species range of hosts for adult ticks.

### *H*, the number of susceptible reservoir hosts

This depends on the host species range and densities of each competent reservoir host species (Tsao, 2009).

### *h*, the host mortality rate

Host mortality rates are intrinsic to each species within the habitats and communities in which they live. However, mortality of hosts does occur that is directly attributable to infection with tick-borne pathogens. Although this is most commonly recognized in domesticated animals, infections of wild animals with tick-borne pathogens can results mortality, and morbidity that may indirectly increase host mortality by increasing vulnerability to other causes of mortality such as predation (Reid et al., 1978). *B. burgdorferi* s.l. is frequently considered non-pathogenic for wild animal hosts such as *P. leucopus* (Wright and Nielsen, 1990). However, there is evidence of pathology associated with infection in juvenile *P. leucopus* (Moody et al., 1994) and in dusky-footed woodrats, *Neotoma fuscipes* (Brown and Lane, 1994). Potential effects of tick-borne pathogens on mortality rates of hosts may be particularly high in newly-invaded regions. Co-evolution of hosts and tick-borne pathogens, resulting in some reductions in pathogen-induced mortality rates, could be expected in endemic regions (Woolhouse et al., 2002). However, immediately after invasion, tick-borne pathogens could have greater impact on mortality rates in pathogen- and tick-naïve host populations. Thus, host mortality rates are intrinsic to the location, and may be increased by invading parasites and pathogens, although to date there is no evidence that this has a significant inhibitory effect on invasion of tick-borne pathogens.

### *r*, the rate at which infected hosts lose their infectivity

This is determined by the individual relationship between the pathogen and host and, for the most part, the rate at which the latter acquires immunity to the former (Ogden et al., 2007; Tsao, 2009). The more persistent host infections are, the more ticks an infected host can infect and most vector-borne pathogens have evolved strategies for persistence in the host by evading the host acquired immune response (Kurtenbach et al., 2006). Some studies suggest that the evolution of investment in acquired immune responses by wild hosts depends on the ‘pace of life’ which is inversely related to their normal life span (Previtali et al., 2012). While this may be a general pattern, there are clearly exceptions. The white-footed mouse *Peromyscus leucopus* can all but eliminate infection with some, but not all, strains of *B. burgdorferi* s.s. suggesting that these mice do acquire immunity to some strains (Hanincová et al., 2008), while *B. burgdorferi* s.s. outer surface genes show evidence of strong balancing selection thought to be driven by host immune responses of the predominanting rodent reservoirs (Qiu et al., 2002). For a particular pathogen at a particular location *r* will, therefore, be determined by the composition of the host community.

As ticks and pathogens become established in new locations and the effective reproduction number (rather than the basic reproduction number) becomes a more meaningful index of the abundance of ticks and pathogens, host acquired immunity (and the capacity of the community overall to acquire immunity/resistance to ticks and pathogens) becomes a more crucial determinant of *N*, *p*^*n*^, *H*, and *h*. The capacity of the host community to express such resistance/immunity will again be a function of the range of species in that community.

In summary, the main factors that determine the capacity for tick and tick-borne pathogen populations to expand and drive invasion, and the suitability of any one location for invasive ticks and tick-borne pathogens to become established are (i) climate (temperature and rainfall); (ii) habitat; and (iii) host species range and density, and (iv) for tick-borne pathogens the presence of, and adaptation to competent vector species. Generalist exophilic tick vectors and generalist pathogens will have greater capacity to invade new regions than specialist ticks and tick-borne pathogens that have a narrow, specialized niche breadth. Climate, habitat and host species range and abundance are highly linked, and together affect suitability for tick invasion; changes in one factor alone is unlikely to drive population or range expansion while one of the other factors is limiting. The rate of range expansion would, however, be expected to vary according to how many limiting factors must change. For example, if habitat and host abundance are suitable, responses to a warming climate may be more rapid than if the warming climate must also drive changes in habitat and host densities for these to become suitable for tick and pathogen invasion. Changes in just one of these factors that are detrimental to tick and tick-borne pathogen persistence would, however, drive range contraction even if the other two factors remain within parameter ranges suitable for tick and tick-borne pathogen persistence. As described above, superimposed on these three main drivers of tick and tick-borne pathogen range change, is the possibility of tick-borne pathogens expanding their populations subsequent to genetic changes that produce novel strains that are capable of exploiting new, or enhancing transmission amongst existing, tick vectors and reservoir host species.

## Capacity for ticks and tick-borne pathogens to be dispersed

### Abundance in source locations

For a location to be a source, the ticks and pathogens must be sufficiently abundant, and the effective reproduction number sufficiently high, that export of ticks and pathogens (i) is precisely balanced by immigration from elsewhere, (ii) is precisely balanced by the rate of reproduction, or (iii) merely releases the ticks and pathogens from density-dependent regulation. If these criteria are not met then ticks and pathogens will die out in the source location. In most circumstances this means that source tick and pathogen populations are undergoing expansion or that tick and pathogen densities are constrained at equilibrium by density-dependent regulation mechanisms.

### Capacity for spread by hosts

Ticks do not fly and are not capable of wind-borne spread so the only way ticks and their pathogens can be dispersed is on or in their hosts. Thus, dispersal is intimately dependent on host movement behavior in dispersal over local scales, and migratory behavior over long distances. In both cases, these may be highly idiosyncratic to the individual locations, tick and pathogen species. Some general patterns can be established regarding long distance dispersal on migratory birds and animals, which may have broadly predictable north-south directions and specific routes through which ticks are dispersed. An example is the dispersion of *I. scapularis* into Canada which has been predicted on the basis of dispersion by migratory passerines (Ogden et al., 2008), and empirical observations support these predictions (Leighton et al., 2012). Northward range expansion of several small mammal species due to climate change (e.g., Myers et al., 2009) may also facilitate vector and pathogen spread. It is possible, however, that climate change may unlink seasonal coincidence of host migration and tick activity as the timing of both could be impacted by changing temperatures (Marra et al., 2005; Ogden et al., 2008) and this may have idiosyncratic impacts of tick and pathogen dispersion (Ogden et al., 2013).

For tick-borne pathogens it has been predicted that dispersal patterns will match those of reservoir hosts. As Lyme borreliosis group spirochetes vary in their host specificity, this hypothesis has been tested utilizing species with narrow reservoir host ranges (see the following and Kurtenbach et al., 2006).

## Expected patterns of tick and tick-borne pathogen genetic diversity in expanding and contracting populations

In the following, our discussion focuses on patterns of genetic diversity of pathogens exemplified by the group of bacteria containing the causative agents of Lyme disease, *B*. *burgdorferi* s.l. Past and present pattern of genetic diversity are likely to be evidenced in genes or sequences showing neutral evolution. Increasingly for *B. burgdorferi* s.l. Multi-Locus Sequence Typing (MLST) of housekeeping genes that show neutral variation is being recognized as a highly useful tool for phylogeographic studies (Margos et al., 2008).

It would be expected that tick and pathogen populations in source locations for range expansion would show genomic evidence of current or past expansion. Evidence of population expansion may be found in mismatch distributions of pairwise nucleotide differences amongst different sequence types/haplotypes in the populations (Schneider and Excoffier, 1999). Adaptive radiation may also be expected when populations expand, although adaptive radiation may lead to host specialization (multiple niche polymorphism), and a narrowing niche for tick and pathogen that may potentially reduce the capacity for dispersion and establishment to new locations or environments (Kempf et al., 2009). Observed patterns of diversity will vary depending on the processes of population expansion, and the time point at which the observation is made. In general, it would be expected that pioneer populations of ticks and tick-borne pathogens at the leading edges of expansion would have constrained genetic diversity, particularly if the new environments are in some way suboptimal, e.g., due to limited or fragmented suitable habitat (Hill et al., 2006). Theoretical studies have suggested that expected patterns of diversity change will depend on whether a range expansion is “pushed,” i.e., the range expansion occurs due to population expansion at the edge of the source location, or “pulled” by pioneers seeding new populations well ahead of the source population range edge (Roques et al., 2012). In the former, expansion is a slower process whereby ecological factors such as the Allee effect constrain population growth allowing diversity in the source population to keep pace with geographic spread. In the latter, genetic diversity in colonizers at the leading edge of range expansion is lower than that in the main body of the population due to stochastic effects of survival of individuals at the colonization front. Long-range dispersal of ticks and tick-borne pathogens by migratory birds may enhance the likelihood that isolated founder populations form the colonization front within which founder effects, i.e., limited genetic diversity, are observed (Mayr, 1963). Skewed diversity may occur at the front of population expansion due to the process of “surfing”; high rates of reproduction at the expanding edge increase mutation rates and new alleles can “surf” the wave of population growth and expand and survive at the leading edge when they may not survive at such abundance, or at all, in a stationary population (Klopfstein et al., 2006). How long and where any of these effects may be seen will depend on the speed at which invasions occur and the degree of connectivity of new populations and the body of the source population (e.g., Banks et al., 2010).

Alongside the northward expansion of the northern limit of the geographic ranges of ticks and tick-borne pathogens with climate change, the southern limit of those ranges may also migrate northwards, i.e., populations at the southern limits may become extinct as climate becomes unsuitable for tick survival (Brownstein et al., 2005; Estrada-Peña and Venzal, 2006). Alternatively, changing environmental conditions may result in changes in tick questing behavior as observed in the Mediterranean for *I. ricinus* (Baptista et al., 2004; Pérez-Eid, 2007) and the southern range of *I. scapularis* (Stromdahl and Hickling, 2012), and adaptation of spirochetes to nidiculous ticks (as perhaps seen in southeastern parts of the US, Lin et al., 2004). Indeed, the consequences of decreasing environmental suitability for ticks and tick-borne pathogens would be expected to be declining diversity because fewer novel genotypes survive, while genotypes that are least resistant to the new environmental stress become extinct (e.g., Wu et al., 2013). The likelihood of extinction will depend on the capacity for ‘evolutionary rescue’ of stressed populations, which in turn depends on the speed of environmental change, immigration rates and the population size, and thus the likelihood that the existing pool of different geno- and phenotypes of ticks and pathogens will contain one or more that is resistant to the changing conditions (Bell, 2013). The severity of the environmental change will also likely determine whether or not refugia may exist within which islands of tick and tick-borne pathogens remain following range contraction. It may, however, be surprising how small such refugia might be. Researchers in Indiana found a 50 ha patch of woodland surrounded by cropland that was sufficient to sustain rodents, *I. scapularis* and *B. burgdorferi* s.s. transmission cycles (Piesman, 2002). Refugial populations could be expected to show divergence from the main population due to genetic drift and narrowed genetic diversity compared to the main population because persistence in likely sub-optimal conditions would be expected to provide a population bottleneck (Provan and Bennett, 2008).

The long term consequences of expansions and contractions of tick and tick-borne pathogen populations would be expected to be reflected, to a greater or lesser degree, in the phylogeography of these organisms. Phylogeography may reveal the genetic relatedness of different genotypes or alleles, directions of ancestry, occurrence and time points at which population events such as expansions and contractions occurred, and how current geographic patterns relate to these observations (Margos et al., 2012). Phylogeography of ticks and tick-borne pathogens is particularly interesting at present because phylogeographic patterns may hold information on effects of past climate changes in the form of glacial and interglacial period cycles, which may predict effects of current anthropogenic climate change on tick and tick-borne pathogen occurrence and diversity (Hofreiter and Stewart, 2009).

## Observed patterns of genetic diversity in ticks and the tick-borne bacterial species complex *borrelia burgdorferi* sensu lato

**Figure 1:**
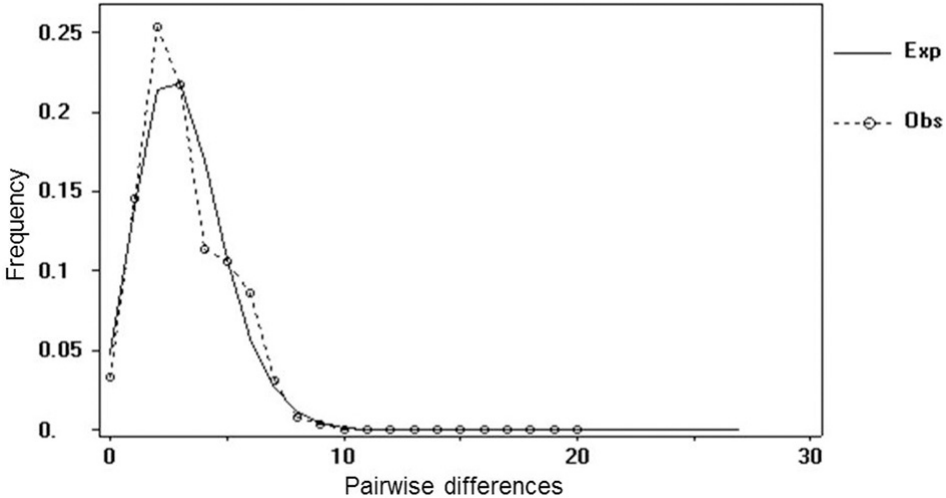
**Pairwise mismatch distribution of 55 mtDNA Cox1 haplotypes of *I. scapularis* in Canada (Obs) compared against the expected distribution for a population under expansion (Exp)**. This graph represents a re-analysis of the data in Mechai et al. (2013) using only one example of each haplotype, to clarify the similarity of observed and expected frequency distributions.

The origin of *B. burgdorferi* s.l. is thought to be Europe (Margos et al., 2008), and consistent with this, the species complex is differentiated in Europe into well-defined species that are frequently host-specialists providing evidence of past adaptive radiation (see below). In contrast, the predominant species in North America is the host generalist *B. burgdorferi* s.s. (Hanincová et al., 2006). Recent expansion of *I. scapularis* and *B. burgdorferi* s.s. in northern USA and into and within Canada provides an opportunity to study what occurs to diversity in tick and pathogen populations undergoing population and range expansions. Pairwise mismatch analysis of mitochondrial gene sequences of *I. scapularis* show the expected evidence of the expansion of northeastern *I. scapularis* populations (Qiu et al., 2002; Figure 1) due to recent environmental changes. In particular, reversal of post-Columbian deforestation by land use changes that permitted re-forestation and expansion of populations of tick hosts (particularly deer) are thought to have driven the expansion of *I. scapularis* and *B. burgdorferi* s.s. populations. This resulted in the Lyme disease epidemic in northern USA that began in the late 1970s, even though some Lyme disease cases had been seen before then (Wood and Lafferty, 2013). Therefore, expansion of *I. scapularis* and *B. burgdorferi* s.s. populations was driven by change in habitat and linked changes in host densities, and occurred within climatically-suitable locations. Increasing deer densities are also likely driving the expansion of *I. ricinus* populations in northern Europe (Medlock et al., 2013). Expansion of populations of *I. scapularis* and *B. burgdorferi* s.s. in north eastern and north central USA, with invasion of new locations, continues today and as ecological conditions (particularly habitat and host abundance) have become more stable, adaptive radiation of *B. burgdorferi* s.s., leading to multiple niche polymorphism, has been suggested (Brisson and Dykhuizen, 2004; Kurtenbach et al., 2006; Brinkerhoff et al., 2010). It is thought that the strain structure of *B. burgdorferi* s.s. may be partly determined by different geographic patterns of tick seasonality. Activity of larval and nymphal *I. scapularis* has greater seasonal coincidence in the north Midwest US compared to the northeastern States (Gatewood et al., 2009) meaning that strains with greater host adaptation may be favored in the northeastern States (Ogden et al., 2007). Studies in Michigan suggest that range expansion of *I. scapularis* in the northern Midwest USA is “pushed” by expanding source populations, although there is evidence of possible refugial *B. burgdorferi* s.s. populations transmitted by nidicolous ticks in locations ahead of expanding *I. scapularis* populations (Hamer et al., 2010).

**Figure 2:**
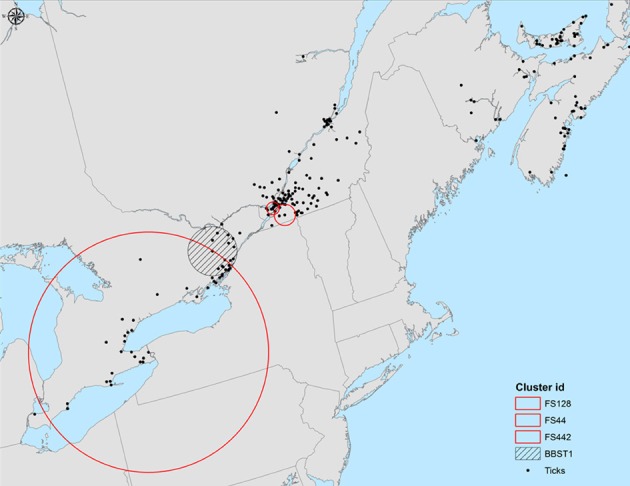
**Spatial cluster analysis of 55 haplotypes of mtDNA Cox1 sequences from *Ixodes scapularis* (see Mechai et al., 2013 for details) and MLST sequence types of *B. burgdorferi* within these ticks collected in passive surveillance in southern Canada**. Three significant clusters of Cox1 haplotypes were identified respectively in south and south-western Quebec and Southern Ontario (red circles). One small-scale cluster of one *B. burgdorferi* ST (ST-01) was also found in south eastern Ontario (hatched circle). Because this ST has been found previously in the northeastern US, and because of the small radius of the cluster, this cluster likely represents a founder event.

**Figure 3:**
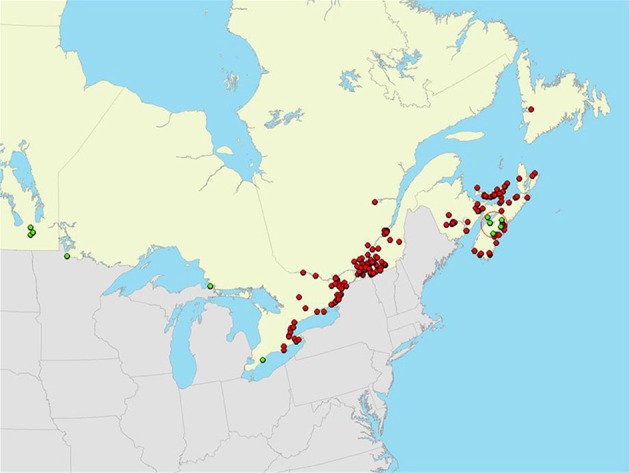
**Geographic distribution of *B. burgdorferi*-infected *Ixodes scapularis* ticks collected in passive surveillance in Canada in which the *ospC* major groups were identified in Ogden et al. (2011)**. A significant spatial cluster of ticks infected with *B. burgdorferi* carrying *ospC* major group I is indicated by the red circle. Reproduced with permission from Ogden et al. (2011).

The *I. scapularis* and *B. burgdorferi* s.s. expansion process now extends to invasion of southern Canada. Analysis of field and surveillance data suggest that invasion of these species is taking place along trajectories determined by all three environmental factors: climate, habitat and host range and abundance (Ogden et al., 2010; Bouchard et al., 2011, 2013; Leighton et al., 2012). In this region the occurrence of small-scale (<30 km radius) spatial clusters of ticks carrying the same mitochondrial gene haplotypes support the hypothesis that *I. scapularis* populations in south eastern Canada have (or have until recently) arisen from isolated founder events (Figure 2). Empirical observations (Ogden et al., 2010; Leighton et al., 2012) are consistent with a hypothesis of climate change-driven range expansion of *I. scapularis* in this region (Ogden et al., 2006b). The occurrence of small-scale clusters of ticks collected in surveillance that carried specific MLST sequence types of *B. burgdorferi* s.s., also support the occurrence of founder populations of *B. burgdorferi s.s*. within founder populations of *I. scapularis* (Figure 2). Evidence for founder events of *B. burgdorferi* s.s. at the geographic scale of a single woodland have been identified by MLST analysis in south eastern Canada (Ogden et al., 2010). Evidence of founder events in the form of clusters of ticks Infected with *B. burgdorferi* s.s. that carried the same allele of the outer surface protein C (*ospC*) gene of *B. burgdorferi* s.s. have also been found in Canada (Figure 3). Although *ospC* is maintained under balancing selection, a high degree of linkage disequilibrium in the northeastern part of the US amongst *B. burgdorferi* s.s. genes suggests that phylogeographic patterns may be partially congruent between *ospC* and MLST, although this may not be the case for all *Borrelia* species and geographic ranges (Barbour and Travinsky, 2010; Hellgren et al., 2011). Together these observations suggest that expansion into Canada is being ‘pulled’ by establishment of founder populations. This supports the hypothesis that recent invasion in this region has occurred via introduction by migratory birds (Ogden et al., 2008) rather than by terrestrial hosts. The Great Lakes and Appalachians have likely posed significant geographic barriers to introduction by terrestrial hosts from the USA to regions of Canada from the Maritimes to Western Ontario. However, the occurrence of one large-scale cluster of ticks carrying the same mitochondrial gene haplotype (radius > 300 km; Figure 2) could provide evidence of a novel genotype ‘surfing’ the rapid growth of *I. scapularis* populations in southern Canada (Leighton et al., 2012), and in the near future range expansion of *I. scapularis* and *B. burgdorferi* s.s. may follow the “pushed” pattern seen in the US with greater importance of terrestrial hosts in tick and pathogen dispersion.

At a continental scale, analysis of MLST data of North American *B. burgdorferi* s.s. populations identified barriers to gene flow amongst northeastern, Midwestern and western populations of *B. burgdorferi* s.s. that are at least partly consistent with our known history of land use changes (Hoen et al., 2009; Margos et al., 2012). MLST data also identify the potential for multiple expansions of *B. burgdorferi* s.s. in North America over a long millennial time scale (Hoen et al., 2009; Margos et al., 2012) that could imply past expansions of *B. burgdorferi* s.s. associated with the onset of climate warming in interglacial periods.

In contrast to the generalist *Borrelia burgdorferi* s.s., which may utilize a diverse range of vertebrates as reservoir hosts including birds and rodents (Hanincová et al., 2006), several European species of the Lyme borreliosis group of spirochetes show distinct pattern of host associations, i.e., the range of reservoir hosts that is able to support completion of the transmission cycle is narrower and consists of either rodent or avian species (reviewed by Kurtenbach et al., 2006). This feature of the ecology of *B. burgdorferi* s.l. in Europe has allowed the hypothesis that host associations determine dispersal rates and geographic patterns of tick-borne pathogens (Vollmer et al., 2011, 2013). These studies showed that populations of the rodent-associated *B. afzelii*, were highly structured and Western and Eastern European populations were identified (Vollmer et al., 2013). The observed pattern of population structure follows that of potential rodent reservoir hosts of *B. afzelii* following the last glacial maximum (Hewitt, 1999; Tougard et al., 2008). Similarly, for *B. lusitaniae*, a species that is transmitted by lizards, distinct populations were found in Portugal south (Grandola) and north (Mafra) of Lisbon using MLST (Vitorino et al., 2008). Different ecological conditions prevail in those habitats and the lizard populations of the Iberian Peninsula are highly geographically structured accordingly (Paulo et al., 2002, 2008). As expected, populations of *Borrelia* species that utilize avian reservoir hosts, such as *B. garinii* and *B*. *valaisiana*, were spatially mixed in Europe and identical MLST sequence types were found in different geographic regions (e.g., England, France, Latvia and Germany) (Vollmer et al., 2011). At a wider continental scale, however, population structure was found even for the bird-associated *Borrelia* species, *B. garinii*, some Asian *B. garinii* sequence types formed a distinct clade in phylogenetic analysis (Vollmer et al., 2013). Additional diversity may be added to *B. garinii* populations via an overlap of marine and terrestrial transmission cycles (Gómez-Díaz et al., 2011).While expansion of *I. scapularis* and *B. burgdorferi* s.s. populations in northeastern US may both show evidence of recent expansion (Qiu et al., 2002), studies have shown no or weak concordance of the phylogeography of these species across the northeastern and Midwestern regions of North America (Humphrey et al., 2010). We have, however, raised the hypothesis that patterns of *Peromyscus* spp. mouse phylogeography may reflect that of *B. burgdorferi* s.s. in North America (Margos et al., 2012). Together these data support the hypothesis that dispersal of tick-borne pathogens is strongly dependent on dispersal behavioral traits of their reservoir hosts.

A further determinant of the phylogeography of *B. burgdorferi* s.l. in Europe is vector competence. In Eastern Europe (from Estonia/Latvia to the Moscow region) populations of *I. ricinis* and *I. persulcatus* (the main Western European vector and the main Asian vector of tick-borne pathogens, respectively) occur sympatrically (Lindquist and Vapalahti, 2008). Certain *B. burgdorferi* s.l. strains are absent in *I. ricinus* (for example NT29 and related strains) while some *Borrelia* species are not or rarely detectable in *I. persulcatus* suggesting vector incompetence for those (Korenberg et al., 2002; Bormane et al., 2004; Masuzawa et al., 2005). The observation that *I. persulcatus* appears to be vector incompetent for *B. burgdorferi* sensu stricto (s.s.) has important implications for historic dispersal and the colonization of the American continent by this species. It suggests that *B. burgdorferi* s.s. and related species either originated in North America (Marti Ras et al., 1997) or invaded the continent from the east (Margos et al., 2008). Also, *B. bavariensis* comprises European and Asian rodent-transmitted strains were previously incorporated into the species *B. garinii* (e.g., Korenberg et al., 2002; Margos et al., 2013; Scholz et al., 2013). However, NT29 and related strains (the Asian variant of *B. bavariensis*) have been associated exclusively with *I. persulcatus* while ospA type 4 (the European variant of *B. bavariensis*) are transmitted by *I. ricinus* (Fingerle et al., 2008). These data suggested that a single clone of *B. bavariensis* adapted to a different vector and was subsequently able to spread into a new geographic region (Margos et al., 2013).

Courtesy of current expansion of *I. scapularis* and *B. burgdorferi* s.s. populations in the USA and Canada we now we have the opportunity to study these processes in real time. Amongst the three key drivers of range change, climate and to some extent habitat are quantifiable, categorisable and measurable allowing some prediction of invasion probabilities (see for example Ogden et al., 2006a,b and Ogden et al., 2008). However, our capacity to predict host species ranges and densities in different locations, and predict host dispersal rates and trajectories, is more limited. Improved knowledge of these factors and their interactions would improve our capacity to develop and calibrate models that predict future patterns of invasion, evolution and diversity of *B. burgdorferi* s.l. associated with current environmental changes. Confidence in such predictions would depend on validation, which is difficult to do prospectively. However, the phylogeographic record may allow us to validate models by hindcasting past population expansions and contractions associated with glacial and interglacial periods, and comparing expected results against currently observed phylogeography. More precise estimates of the molecular clock of selectively neutral loci would be particularly useful for this purpose.

## Conclusion

Here we have outlined processes involved in the expansion and invasion of tick and tick-borne pathogen populations and identified changes in climate, habitat and hosts as key factors in range changes for ticks and tick-borne diseases. Host movements are the main mechanism of dispersal of ticks and tick-borne pathogens that permits invasions. We have also reviewed expected patterns of diversity of ticks and tick-borne pathogens in zones of emergence and geographic spread and we identify that for the Lyme borreliosis spirochaetes and vectors many observed patterns are consistent with the patterns expected from theoretical studies or studies of other species. Studies of the agent of Lyme disease *B. burgdorferi* s.l. suggest that the phylogeographic and ancestral patterns held within the genome of tick-borne pathogens may depend closely on host behavior and dispersion, and provide clues as to how their populations expanded and contracted associated with past climatic changes. Study of host dispersion and current range expansions may help us to develop better predictive models of effects of current climate change on patterns of invasion and diversity of ticks and tick-borne pathogens, while the phylogeographic record of past population expansions and contractions may allow us to validate these models.

### Conflict of interest statement

The authors declare that the research was conducted in the absence of any commercial or financial relationships that could be construed as a potential conflict of interest.

## References

[B1] Banks S. C. Ling S. D. Johnson C. R. Piggott M. P. Williamson J. E. Beheregaray L. B. (2010). Genetic structure of a recent climate change-driven range extension. Mol. Ecol. 19, 2011–2024 10.1111/j.1365-294X.2010.04627.x 20406383

[B2] Baptista S. Quaresma A. Aires T. Kurtenbach K. Santos-Reis M. Nicholson M. (2004). Lyme borreliosis spirochetes in questing ticks from mainland Portugal. Int. J. Med. Microbiol. 293, 109–116 15146992 10.1016/s1433-1128(04)80016-0

[B3] Barbour A. G. Travinsky B. (2010). Evolution and distribution of the ospC gene, a transferable serotype determinant of *Borrelia burgdorferi*. MBio 1:e00153–00110 20877579 10.1128/mBio.00153-10 PMC2945197

[B4] Begon M. Townsend C. R. Harper J. L. (2005). Ecology: From Individuals to Ecosystems, 4th Edn. Hoboken: Wiley-Blackwell

[B5] Bell G. (2013). Evolutionary rescue and the limits of adaptation. Philos. Trans. R. Soc. Lond. B Biol. Sci. 368, 20120080 10.1098/rstb.2012.0080 23209162 PMC3538447

[B6] Bormane A. Lucenko I. Duks A. Mavtchoutko V. Ranka R. Salmina K. (2004). Vectors of tick-borne diseases and epidemiological situation in Latvia in 1993–2002. Int. J. Med. Microbiol. 293, 36–47 15146983 10.1016/s1433-1128(04)80007-x

[B7] Bouchard C. Beauchamp G. Nguon S. Trudel L. Milord F. Lindsay L. R. (2011). Associations between Ixodes scapularis ticks and small mammal hosts in a newly-endemic zone in southeastern Canada: implications for *Borrelia burgdorferi* transmission. Ticks Tick Borne Dis. 2, 183–190 10.1016/j.ttbdis.2011.03.005 22108010

[B8] Bouchard C. Leighton P. Beauchamp G. Nguon S. Trudel L. Milord F. (2013). Harvested white-tailed deer as sentinel hosts for early establishing *Ixodes scapularis* populations and risk from vector-borne zoonoses in southeastern Canada. J. Med. Entomol. 50, 384–393 10.1603/ME12093 23540128

[B9] Brinkerhoff R. J. Bent S. J. Folsom-O'Keefe C. M. Tsao K. Hoen A. G. Barbour A. G. (2010). Genotypic diversity of *Borrelia burgdorferi* strains detected in *Ixodes scapularis* larvae collected from North American songbirds. Appl. Environ. Microbiol. 76, 8265–8268 10.1128/AEM.01585-10 20971869 PMC3008240

[B10] Brisson D. Dykhuizen D. E. (2004). *ospC* diversity in *Borrelia burgdorferi*: different hosts are different niches. Genetics 168, 713–722 10.1534/genetics.104.028738 15514047 PMC1448846

[B11] Brown R. N. Lane R. S. (1994). Natural and experimental Borrelia burgdorferi infections in woodrats and deer mice from California. J. Wildl. Dis. 30, 389–398 7933283 10.7589/0090-3558-30.3.389

[B12] Brownstein J. S. Holford T. R. Fish D. (2005). Effect of climate change on Lyme disease risk in North America. Ecohealth 2, 38–46 10.1007/s10393-004-0139-x 19008966 PMC2582486

[B13] Dizij A. Kurtenbach K. (1995). Clethrionomys glareolus, but not Apodemus flavicollis, acquires resistance to Ixodes ricinus L., the main European vector of *Borrelia burgdorferi*. Parasite Immunol. 17, 177–183 10.1111/j.1365-3024.1995.tb00887.x 7624158

[B14] Dobson A. Cattadori I. Holt R. D. Ostfeld R. S. Keesing F. Krichbaum K. (2006). Sacred cows and sympathetic squirrels: the importance of biological diversity to human health. PLoS Med. 3:e231 10.1371/journal.pmed.0030231 16729846 PMC1472550

[B15] Estrada-Peña A. Venzal J. M. (2006). Changes in habitat suitability for the tick *Ixodes ricinus* (Acari: Ixodidae) in Europe (1900–1999). EcoHealth 3, 154–162 10.1007/s10393-006-0036-6

[B16] Fingerle V. Schulte-Spechtel U. C. Ruzic-Sabljic E. Leonhard S. Hofmann H. Weber K. (2008). Epidemiological aspects and molecular characterization of *Borrelia burgdorferi* sl. from southern Germany with special respect to the new species *Borrelia spielmanii* sp. nov. Int. J. Med. Microbiol. 298, 279–290 10.1016/j.ijmm.2007.05.002 17616434

[B18] Gatewood A. G. Liebman K. A. Vourc'h G. Bunikis J. Hamer S. A. Cortinas R. (2009). Climate and tick seasonality are predictors of *Borrelia burgdorferi* genotype distribution. Appl. Environ. Microbiol. 75, 2476–2483 10.1128/AEM.02633-08 19251900 PMC2675205

[B19] Gómez-Díaz E. Boulinier T. Sertour N. Cornet M. Ferquel E. McCoy K. D. (2011). Genetic structure of marine *Borrelia garinii* and population admixture with the terrestrial cycle of *Lyme borreliosis*. Environ. Microbiol. 13, 2453–2467 10.1111/j.1462-2920.2011.02515.x 21651685

[B20] Goodwin B. J. Ostfeld R. S. Schauber E. M. (2001). Spatiotemporal variation in a Lyme disease host and vector: black-legged ticks on white-footed mice. Vector Borne Zoonotic Dis. 1, 129–138 10.1089/153036601316977732 12653143

[B21] Gray J. S. (1991). The development and seasonal activity of the tick *Ixodes ricinus*: a vector of Lyme borreliosis. Rev. Med. Vet. Entomol. 79, 323–333

[B22] Hamer S. A. Tsao J. I. Walker E. D. Hickling G. J. (2010). Invasion of the Lyme disease vector *Ixodes scapularis*: implications for *Borrelia burgdorferi* endemicity. Ecohealth 7, 47–63 10.1007/s10393-010-0287-0 20229127

[B23] Hanincová K. Kurtenbach K. Diuk-Wasser M. Brei B. Fish D. (2006). Epidemic spread of *Lyme borreliosis*, northeastern United States. Emerg. Infect. Dis. 12, 604–611 10.3201/eid1204.051016 16704808 PMC3294694

[B24a] Hanincová K. Ogden N. H. Diuk-Wasser M. Pappas C. J. Iyer R. Fish D. (2008). Fitness variation of *Borrelia burgdorferi* sensu stricto strains in mice. Appl. Environ. Microbiol. 74, 153–157 10.1128/AEM.01567-07 17981941 PMC2223198

[B24] Hellgren O. Andersson M. Raberg L. (2011). The genetic structure of *Borrelia afzelii* varies with geographic but not ecological sampling scale. J. Evol. Biol. 24, 159–167 10.1111/j.1420-9101.2010.02148.x 20964784

[B25] Hewitt G. M. (1999). Post-glacial re-colonization of European biota. Biol. J. Linnean Soc. 68, 87–112 10.1111/j.1095-8312.1999.tb01160.x19002203

[B26] Hill J. K. Hughes C. L. Dytham C. Searle J. B. (2006). Genetic diversity in butterflies: interactive effects of habitat fragmentation and climate-driven range expansion. Biol. Lett. 2, 152–154 10.1098/rsbl.2005.0401 17148351 PMC1617171

[B27] Hoen A. G. Margos G. Bent S. J. Diuk-Wasser M. A. Barbour A. Kurtenbach K. (2009). Phylogeography of *Borrelia burgdorferi* in the eastern United States reflects multiple independent Lyme disease emergence events. Proc. Natl. Acad. Sci. U.S.A. 106, 15013–15018 10.1073/pnas.0903810106 19706476 PMC2727481

[B28] Hofreiter M. Stewart J. (2009). Ecological change, range fluctuations and population dynamics during the Pleistocene. Curr. Biol. 19, R584–R594 10.1016/j.cub.2009.06.030 19640497

[B29] Humphrey P. T. Caporale D. A. Brisson D. (2010). Uncoordinated phylogeography of *Borrelia burgdorferi* and its tick vector, *Ixodes scapularis*. Evolution 64, 2653–2663 10.1111/j.1558-5646.2010.01001.x 20394659 PMC2919648

[B30] Keesing F. Brunner J. Duerr S. Killilea M. Logiudice K. Schmidt K. (2009). Hosts as ecological traps for the vector of Lyme disease. Proc. Biol. Sci. 276, 3911–3919 10.1098/rspb.2009.1159 19692412 PMC2825780

[B31] Keesing F. Holt R. D. Ostfeld R. S. (2006). Effects of species diversity on disease risk. Ecol. Lett. 9, 485–498 10.1111/j.1461-0248.2006.00885.x 16623733

[B32] Kempf F. Boulinier T. de Meeûs T. Arnathau C. McCoy K. D. (2009). Recent evolution of host-associated divergence in the seabird tick *Ixodes uriae*. Mol. Ecol. 18, 4450–4462 10.1111/j.1365-294X.2009.04356.x 19793353

[B33] Kilpatrick A. M. Randolph S. E. (2012). Drivers, dynamics, and control of emerging vector-borne zoonotic diseases. Lancet 380, 1946–1955 10.1016/S0140-6736(12)61151-9 23200503 PMC3739480

[B34] Klopfstein S. Currat M. Excoffier L. (2006). The fate of mutations surfing on the wave of a range expansion Mol. Biol. Evol. 23, 482–490 10.1093/molbev/msj057 16280540

[B35] Korenberg E. I. Gorelova N. B. Kovalevskii Y. (2002). Ecology of Borrelia burgdorferi sensu lato in Russia, in Lyme Borreliosis: Biology, Epidemiology and Control, eds Gray J. S. Kahl O. Lane R. S. Stanek G. (Wallingford: CABI Publishing), 175–200 10.1079/9780851996325.0175

[B36] Kurtenbach K. De Michelis S. Etti S. Schäfer S. M. Sewell H. S. Brade V. (2002). Host association of *Borrelia burgdorferi* sensu lato–the key role of host complement. Trends Microbiol. 10, 74–79 10.1016/S0966-842X(01)02298-3 11827808

[B37] Kurtenbach K. Hanincová K. Tsao J. Margos G. Fish D. Ogden N. H. (2006). Key processes in the evolutionary ecology of *Lyme borreliosis*. Nat. Rev. Microbiol. 4, 660–669 10.1038/nrmicro1475 16894341

[B38] Léger E. Vourc'h G. Vial L. Chevillon C. McCoy K. D. (2013). Changing distributions of ticks: causes and consequences. Exp. Appl. Acarol. 59, 219–244 10.1007/s10493-012-9615-0 23015121

[B39] Leighton P. Koffi J. Pelcat Y. Lindsay L. R. Ogden N. H. (2012). Predicting the speed of tick invasion: an empirical model of range expansion for the Lyme disease vector *Ixodes scapularis* in Canada. J. Appl. Ecol. 49, 457–464 10.1111/j.1365-2664.2012.02112.x

[B40] Levin M. L. Fish D. (1998). Density-dependent factors regulating feeding success of *Ixodes scapularis* larvae (Acari: Ixodidae). J. Parasitol. 84, 36–43 10.2307/3284526 9488335

[B41] Lin T. Oliver J. H. Jr Gao L. (2004). Molecular characterization of *Borrelia* isolates from ticks and mammals from the southern United States. J. Parasitol. 90, 1298–1307 10.1645/GE-195R1 15715220

[B42] Lindgren E. Tälleklint L. Polfeldt T. (2000). Impact of climatic change on the northern latitude limit and population density of the disease-transmitting European tick *Ixodes ricinus*. Environ. Health Perspect. 108, 119–123 10.1289/ehp.00108119 10656851 PMC1637900

[B43] Lindsay L. R. Barker I. K. Surgeoner G. A. McEwen S. A. Gillespie T. J. Addison E. M. (1998). Survival and development of the different life stages of *Ixodes scapularis* (Acari: Ixodidae) held within four habitats on Long Point, Ontario, Canada. J. Med. Entomol. 35, 189–199 9615533 10.1093/jmedent/35.3.189

[B44] Lindsay L. R. Barker I. K. Surgeoner G. A. McEwen S. A. Gillespie T. J. Robinson J. T. (1995). Survival and development of *Ixodes scapularis* (Acari: Ixodidae) under various climatic conditions in Ontario, Canada. J. Med. Entomol. 32, 143–152 7608920 10.1093/jmedent/32.2.143

[B45] Lindquist L. Vapalahti O. (2008). Tick-borne encephalitis. Lancet 371, 1861–1871 10.1016/S0140-6736(08)60800-4 18514730

[B46] Margos G. Gatewood A. G. Aanensen D. M. Hanincová K. Terekhova D. Vollmer S. A. (2008). MLST of housekeeping genes captures geographic population structure and suggests a European origin of *Borrelia burgdorferi*. Proc. Natl. Acad. Sci. U.S.A. 105, 8730–8735 10.1073/pnas.0800323105 18574151 PMC2435589

[B47] Margos G. Tsao J. I. Castillo-Ramírez S. Girard Y. A. Hamer S. A. Hoen A. G. (2012). Two boundaries separate *Borrelia burgdorferi* populations in North America. Appl. Environ. Microbiol. 78, 6059–6067 10.1128/AEM.00231-12 22729536 PMC3416618

[B49] Margos G. Wilske B. Sing A. Hizo-Teufel C. Cao W.-C. Chu C. (2013). *Borrelia bavariensis* sp. nov. is widely distributed in Europe and Asia. Int. J. Syst. Evol. Microbiol. [Epub ahead of print]. 10.1099/ijs.0.052001-0 23838444

[B50] Marra P. P. Francis C. M. Mulvihill R. S. Moore F. R. (2005). The influence of climate on the timing and rate of spring bird migration. Oecologia 142, 307–315 10.1007/s00442-004-1725-x 15480801

[B51] Marti Ras N. Postic D. Foretz M. Baranton G. (1997). *Borrelia burgdorferi* sensu stricto, a bacterial species “made in the U.S.A.”. Int. J. Syst. Bacteriol. 47, 1112–1117 10.1099/00207713-47-4-1112 9336916

[B52] Masuzawa T. Kharitonenkov I. G. Kadosaka T. Hashimoto N. Kudeken M. Takada N. (2005). Characterization of *Borrelia burgdorferi* sensu lato isolated in Moscow province - a sympatric region for *Ixodes ricinus* and *Ixodes persulcatus*. Int. J. Med. Microbiol. 294, 455–464 10.1016/j.ijmm.2004.09.008 15715174

[B53] Mayr E. (1963). Animal Species and Evolution. Cambridge: Harvard University Press

[B54a] Mechai S. Feil E. J. Gariepy T. D. Gregory T. R. Lindsay L. R. Millien V. (2013). Investigation of the population structure of the tick vector of Lyme disease *Ixodes scapularis* (Acari: *Ixodidae*) in Canada using mitochondrial cytochrome C oxidase subunit I gene sequences. J. Med. Entomol. 50, 560–570 10.1603/ME12178 23802450

[B54] Medlock J. M. Hansford K. M. Bormane A. Derdakova M. Estrada-Pena A. George J. C. (2013). Driving forces for changes in geographical distribution of *Ixodes ricinus* ticks in Europe. Parasit. Vectors 6:1 10.1186/1756-3305-6-1 23281838 PMC3549795

[B55] Moody K. D. Terwilliger G. A. Hansen G. M. Barthold S. W. (1994). Experimental *Borrelia bu*rgdorferi infection in *Peromyscus leucopus*. J. Wildl. Dis. 30, 155–161 8028098 10.7589/0090-3558-30.2.155

[B56] Myers P. Lundrigan B. L. Hoffman S. M. G. Poor Haraminac A. Seto S. H. (2009). Climate-induced changes in small mammal communities of the Northern Great Lakes Region. Glob. Change Biol. 15, 1434–1454 10.1111/j.1365-2486.2009.01846.x

[B57] Norman R. Bowers R. G. Begon M. Hudson P. J. (1999). Persistence of tick-borne virus in the presence of multiple host species: tick reservoirs and parasite mediated competition. J. Theor. Biol. 200, 111–118 10.1006/jtbi.1999.0982 10479543

[B59] Ogden N. H. Barker I. K. Beauchamp G. Brazeau S. Charron D. Maarouf A. (2006a). Investigation of ground level and remote-sensed data for habitat classification and prediction of survival of *Ixodes scapularis* ticks in habitats of southeastern Canada. J. Med. Entomol. 43, 403–414 10.1603/0022-2585(2006)043[0403:IOGLAR]2.0.CO;2 16619627

[B60] Ogden N. H. Maarouf A. Barker I. K. Bigras-Poulin M. Lindsay L. R. Morshed M. G. (2006b). Climate change and the potential for range expansion of the Lyme disease vector *Ixodes scapularis* in Canada. Int. J. Parasitol. 36, 63–70 10.1016/j.ijpara.2005.08.016 16229849

[B61] Ogden N. H. Bigras-Poulin M. O'Callaghan C. J. Barker I. K. Lindsay L. R. Maarouf A. (2005). A dynamic population model to investigate effects of climate on geographic range and seasonality of the tick *Ixodes scapularis*. Int. J. Parasitol. 35, 375–389 10.1016/j.ijpara.2004.12.013 15777914

[B62] Ogden N. H. Bigras-Poulin M. O'Callaghan C. J. Barker I. K. Lindsay L. R. Maarouf A. (2007). Tick seasonality, host infection dynamics and fitness of *Ixodes scapularis*-borne pathogens. Parasitology. 134, 209–227 10.1017/S0031182006001417 17032476

[B63] Ogden N. H. Bouchard C. Kurtenbach K. Margos G. Lindsay L. R. Trudel L. (2010). Active and passive surveillance, and phylogenetic analysis of *Borrelia burgdorferi* elucidate the process of Lyme disease risk emergence in Canada. Environ. Health Perspect. 118, 909–914 10.1289/ehp.0901766 20421192 PMC2920908

[B64] Ogden N. H. Casey A. N. J. French N. P. Adams J. D. W. Woldehiwet Z. (2002a). Field evidence for density-dependent facilitation amongst Ixodes ricinus ticks feeding on sheep. Parasitology 124, 117–125 10.1017/S0031182001001081 11862990

[B65] Ogden N. H. Casey A. N. J. Lawrie C. French N. P. Woldehiwet Z. Carter S. D. (2002b). IgG responses to salivary gland extract of Ixodes ricinus ticks vary inversely with resistance in naturally exposed sheep. Med. Vet. Entomol. 16, 186–192 10.1046/j.1365-2915.2002.00362.x 12109713

[B66] Ogden N. H. Lindsay R. L. Hanincová K. Barker I. K. Bigras-Poulin M. Charron D. F. (2008). The role of migratory birds in introduction and range expansion of *Ixodes scapularis* ticks, and *Borrelia burgdorferi* and *Anaplasma phagocytophilum* in Canada. Appl. Environ. Microbiol. 74, 1780–1790 10.1128/AEM.01982-07 18245258 PMC2268299

[B67] Ogden N. H. Lindsay L. R. Leighton P. (2013). Predicting the rate of invasion of the agent of Lyme disease, *Borrelia burgdorferi* in North America. J. Appl. Ecol. 50, 510–518 10.1111/1365-2664.12050

[B68] Ogden N. H. Lindsay R. L. Sockett P. N. Morshed M. Artsob H. (2009). Emergence of Lyme disease in Canada. CMAJ 180, 1221–1224 10.1503/cmaj.080148 19506281 PMC2691438

[B69] Ogden N. H. Margos G. Aanensen D. M. Drebot M. A. Feil E. J. Hanincová K. (2011). Investigation of genotypes of *Borrelia burgdorferi* in *Ixodes scapularis* ticks collected in surveillance in Canada. Appl. Environ. Microbiol. 77, 3244–3254 10.1128/AEM.02636-10 21421790 PMC3126474

[B70] Ogden N. H. Tsao J. I. (2009). Biodiversity and Lyme disease: dilution or amplification. Epidemics 1, 196–206 10.1016/j.epidem.2009.06.002 21352766

[B71] Ostfeld R. S. Keesing F. (2000). Biodiversity and disease risk: the case of Lyme disease. Conserv. Biol. 14, 722–728 10.1046/j.1523-1739.2000.99014.x21652151

[B72] Paulo O. S. Jordan W. C. Bruford M. W. Nichols R. A. (2002). Using nested clade analysis to assess the history of colonization and the persistence of populations of an Iberian lizard. Mol. Ecol. 11, 809–819 10.1046/j.1365-294X.2002.01484.x 11972766

[B73] Paulo O. S. Pinheiro J. Miraldo A. Bruford M. W. Jordan W. C. Nichols R. A. (2008). The role of vicariance vs. dispersal in shaping genetic patterns in ocellated lizard species in the western Mediterranean. Mol. Ecol. 17, 1535–1551 10.1111/j.1365-294X.2008.03706.x 21928468

[B74] Pérez-Eid C. (2007). Les tiques: Identification, biologie, importance médicale et vétérinaire. Paris: Tec and Doc Lavoisier

[B75] Piesman J. (2002). Ecology of Borrelia burgdorferi sensu lato in North America, in Lyme Borreliosis: Biology, Epidemiology and Control, eds Gray J. S. Kahl O. Lane R. S. Stanek G. (Wallingford: CABI Publishing), 223–250 10.1079/9780851996325.0223

[B76] Previtali M. A. Ostfeld R. S. Keesing F. Jolles A. E. Hanselmann R. Martin L. B. (2012). Relationship between pace of life and immune responses in wild rodents. Oikos 121, 1483–1492 10.1111/j.1600-0706.2012.020215.x

[B77] Provan J. Bennett K. D. (2008). Phylogeographic insights into cryptic glacial refugia. Trends Ecol. Evol. 23, 564–571 10.1016/j.tree.2008.06.010 18722689

[B78] Qiu W. G. Dykhuizen D. E. Acosta M. S. Luft B. J. (2002). Geographic uniformity of the Lyme disease spirochete (*Borrelia burgdorferi*) and its shared history with tick vector (*Ixodes scapularis*) in the Northeastern United States. Genetics 160, 833–849 11901105 10.1093/genetics/160.3.833 PMC1462027

[B79] Randolph S. E. (1979). Population regulation in ticks: the role of acquired resistance in natural and unnatural hosts. Parasitology 79, 141–156 10.1017/S0031182000052033 542316

[B80] Randolph S. E. (1997). Abiotic and biotic determinants of the seasonal dynamics of the tick *Rhipicephalus appendiculatus* in South Africa. Med. Vet. Entomol. 11, 25–37 10.1111/j.1365-2915.1997.tb00286.x 9061674

[B81] Randolph S. E. (1998). Ticks are not insects: consequences of contrasting vector biology for transmission potential. Parasitol. Today 14, 186–192 10.1016/S0169-4758(98)01224-1 17040748

[B82] Randolph S. E. Gern L. Nuttall P. A. (1996). Co-feeding ticks: epidemiological significance for tick-borne pathogen transmission. Parasitol. Today 12, 472–479 10.1016/S0169-4758(96)10072-7 15275266

[B83] Randolph S. E. Green R. M. Hoodless A. N. Peacey M. F. (2002). An empirical quantitative framework for the seasonal population dynamics of the tick *Ixodes ricinus*. Int. J. Parasitol. 32, 979–989 10.1016/S0020-7519(02)00030-9 12076627

[B84] Randolph S. E. Miklisová D. Lysy J. Rogers D. J. Labuda M. (1999). Incidence from coincidence: patterns of tick infestations on rodents facilitate transmission of tick-borne encephalitis virus. Parasitology 118, 177–186 10.1017/S0031182098003643 10028532

[B85] Reid H. W. Duncan J. S. Phillips J. D. Moss R. Watson A. (1978). Studies of louping-ill virus (Flavivirus group) in wild red grouse (*Lagopus lagopus scoticus*). J. Hyg. (Lond.) 81, 321–329 10.1017/S002217240002516X 212479 PMC2129774

[B86] Roques L. Garnier J. Hamel F. Klein E. K. (2012). Allee effect promotes diversity in traveling waves of colonization. Proc. Natl. Acad. Sci. U.S.A. 109, 8828–8833 10.1073/pnas.1201695109 22611189 PMC3384151

[B87] Schneider S. Excoffier L. (1999). Estimation of past demographic parameters from the distribution of pairwise differences when the mutation rates vary among sites: application to human mitochondrial DNA. Genetics 152, 1079–1089 10388826 10.1093/genetics/152.3.1079 PMC1460660

[B88] Scholz H. C. Margos G. Derschum H. Speck S. Tserennorov D. Erdenebat N. (2013). High prevalence of genetically diverse *Borrelia bavariensis*-like strains in Ixodes persulcatus from Selenge Aimag, Mongolia. Ticks Tick Borne Dis. 4, 89–92 10.1016/j.ttbdis.2012.08.004 23084366

[B89] Shaw M. T. Keesing F. McGrail R. Ostfeld R. S. (2003). Factors influencing the distribution of larval blacklegged ticks on rodent hosts. Am. J. Trop. Med. Hyg. 68, 447–452 12875294

[B90] Stromdahl E. Y. Hickling G. J. (2012). Beyond Lyme: aetiology of Tick-borne human diseases with emphasis on the South-Eastern United States. Zoonoses Public Health 59, 48–64 10.1111/j.1863-2378.2012.01475.x 22958250

[B91] Tougard C. Renvoisé E. Petitjean A. Quéré J.-P. (2008). New insight into the colonization processes of common voles: inferences from molecular and fossil evidence. PLoS ONE 3:e3532 10.1371/journal.pone.0003532 18958287 PMC2570793

[B92] Tsao J. I. (2009). Reviewing molecular adaptations of Lyme borreliosis spirochetes in the context of reproductive fitness in natural transmission cycles. Vet. Res. 40, 36 10.1051/vetres/2009019 19368764 PMC2701186

[B93] Vail S. C. Smith G. J. (1998). Air temperature and relative humidity effects on behavioral activity of blacklegged tick (Acari: Ixodidae) nymphs in New Jersey. J. Med. Entomol. 35, 1025–1028 9835697 10.1093/jmedent/35.6.1025

[B94] Vail S. C. Smith G. J. (2002). Vertical movement and posture of blacklegged tick (Acari: Ixodidae) nymphs as a function of temperature and relative humidity in laboratory experiments. J. Med. Entomol. 39, 842–846 10.1603/0022-2585-39.6.842 12495181

[B95] Vitorino L. R. Margos G. Feil E. J. Collares-Pereira M. Zé-Zé L. Kurtenbach K. (2008). Fine-scale phylogeographic structure of *Borrelia lusitaniae* revealed by Multilocus Sequence Typing. PLoS ONE 3:e4002 10.1371/journal.pone.0004002 19104655 PMC2602731

[B96] Vollmer S. A. Bormane A. Dinnis R. E. Seelig F. Dobson A. D. Aanensen D. M. (2011). Host migration impacts on the phylogeography of *Lyme Borreliosis* spirochaete species in Europe. Environ. Microbiol. 13, 184–192 10.1111/j.1462-2920.2010.02319.x 20722696

[B97] Vollmer S. A. Feil E. J. Chu C. Y. Raper S. L. Cao W. C. Kurtenbach K. (2013). Spatial spread and demographic expansion of *Lyme borreliosis* spirochaetes in Eurasia. Infect. Genet. Evol. 14, 147–155 10.1016/j.meegid.2012.11.014 23219915

[B98a] Wikel S. K. (1999). Tick modulation of host immunity: an important factor in pathogen transmission. Int. J. Parasitol. 29, 851–859 10.1016/S0020-7519(99)00042-9 10480722

[B98] Wood C. L. Lafferty K. D. (2013). Biodiversity and disease: a synthesis of ecological perspectives on Lyme disease transmission. Trends Ecol. Evol. 28, 239–247 10.1016/j.tree.2012.10.011 23182683

[B99] Woolhouse M. E. J. Webster J. P. Domingo E. Charlesworth B. Levin B. R. (2002). Biological and biomedical implications of the coevolution of pathogens and their hosts. Nat. Genet. 32, 569–577 10.1038/ng1202-569 12457190

[B100] Wright S. D. Nielsen S. W. (1990). Experimental infection of the white-footed mouse with *Borrelia burgdorferi*. Am. J. Vet. Res. 5, 1980–1987 2085225

[B101] Wu Y. Wang Y. Jiang K. Hanken J. (2013). Significance of pre-Quaternary climate change for montane species diversity: insights from Asian salamanders (Salamandridae: Pachytriton). Mol. Phylogenet. Evol. 66, 380–390 10.1016/j.ympev.2012.10.011 23110935

